# SARS-CoV-2 Omicron Symptomatic Infections in Previously Infected or Vaccinated South African Healthcare Workers

**DOI:** 10.3390/vaccines10030459

**Published:** 2022-03-17

**Authors:** Marta C. Nunes, Sthembile Mbotwe-Sibanda, Vicky L. Baillie, Gaurav Kwatra, Ricardo Aguas, Shabir A. Madhi, on behalf of the Wits VIDA HCW Study Group

**Affiliations:** 1 South African Medical Research Council, Vaccines and Infectious Diseases Analytics Research Unit, Faculty of Health Sciences, University of the Witwatersrand, Johannesburg 1862, South Africa; sthembile.sibanda@wits-vida.org (S.M.-S.); vicky.baillie@wits-vida.org (V.L.B.); gaurav.kwatra@wits-vida.org (G.K.); shabir.madhi@wits.ac.za (S.A.M.); 2 Department of Science and Technology/National Research Foundation: Vaccine Preventable Diseases Unit, Faculty of Health Sciences, University of the Witwatersrand, Johannesburg 1862, South Africa; 3 Centre for Tropical Medicine and Global Health, Nuffield Department of Medicine, University of Oxford, Oxford OX1 3SY, UK; ricardo@tropmedres.ac; 4 African Leadership in Vaccinology Expertise, Faculty of Health Sciences, University of the Witwatersrand, Johannesburg 2193, South Africa

**Keywords:** COVID-19, SARS-CoV-2, Omicron, reinfection, vaccination

## Abstract

We investigated Omicron infections among healthcare workers (HCW) presenting with symptoms of SARS-CoV-2 infection and evaluated the protective effect of vaccination or prior infection. Between 24 November and 31 December 2021, HCW in Johannesburg, South Africa, were tested for SARS-CoV-2 infection by Nucleic Acid Amplification Test (NAAT). Blood samples collected either at the symptomatic visit or in the 3 months prior, were tested for spike protein immunoglobulin G (IgG). Overall, 433 symptomatic HCW were included in the analysis, with 190 (43.9%) having an Omicron infection; 69 (16.7%) were unvaccinated and 270 (62.4%) received a single dose of the Ad26.COV.2 vaccine. There was no difference in the odds of identifying Omicron between unvaccinated and Ad26.COV.2 vaccinated HCW (adjusted odds ratio (aOR) 0.81, 95% confidence interval (CI): 0.46, 1.43). One-hundred and fifty-four (35.3%) HCW had at least one SARS-CoV-2 NAAT-confirmed prior infection; these had lower odds of Omicron infection compared with those without past infection (aOR 0.55, 95%CI: 0.36, 0.84). Anti-spike IgG concentration of 1549 binding antibody unit/mL was suggestive of significant reduction in the risk of symptomatic Omicron infection. We found high reinfection and vaccine breakthrough infection rates with the Omicron variant among HCW. Prior infection and high anti-spike IgG concentration were protective against Omicron infection.

## 1. Introduction

The Omicron (B.1.1.529/21K) severe acute respiratory syndrome coronavirus-2 (SARS-CoV-2) variant was reported in South Africa on 25 November 2021, following investigation of a rapid increase in coronavirus disease (COVID-19) cases in the Gauteng province, and identification of a spike gene target failure (SGTF) on the Taqpath assay (ThermoFisher™), which also includes two other gene targets [1]. The Omicron variant has now been described to contain mutations that confer more infectious (double mutation in nucleocapsid, R203K, G204R), more transmissible (H655Y, N679K, P681H mutations in the spike protein), and higher ability to evade host immunity (including ∆105–107 mutation affecting nonstructural proteins and multiple other mutations affecting the spike protein receptor binding domain (RBD) and N-terminal domain) compared with the ancestral virus [1,2]. In December 2021, the Omicron variant constituted 98% of all SARS-CoV-2 infections in South Africa, and has now spread globally [3].

Healthcare workers (HCW) in South Africa were offered the Ad26.COV.2 COVID-19 vaccine as part of the Sisonke trial from 17 February 2021 as a single dose schedule, and subsequently, a booster dose was offered since 8 November 2021 [4,5]. From May 2021, HCW could also access the BNT162b2 COVID-19 vaccine as part of the national vaccine rollout in South Africa.

Here, we describe the Omicron infections among HCW who presented with symptoms suggestive of SARS-CoV-2 infection from 24 November to 31 December 2021. We also detailed breakthrough infections in vaccinated HCW and reinfections in previous Nucleic Acid Amplification Test (NAAT)-confirmed SARS-CoV-2 cases. In addition, blood samples collected either at the symptomatic visit or in the 3 months prior were tested for full-length SARS-CoV-2 spike protein immunoglobulin G (IgG) to assess the potential protective effect of these antibodies against Omicron infection.

## 2. Methods

### 2.1. Study Design

Healthcare workers (HCW) working at the Chris Hani Baragwanath Academic Hospital (CHBAH) in Johannesburg, Gauteng province, South Africa were enrolled from April to July 2020, into a longitudinal cohort surveillance study; this cohort has been previously described [6]. Due to participants discontinuing the study, enrolments into the longitudinal cohort were restarted on 16 February 2021 and closed on 10 August 2021. Among the longitudinal cohort participants, routine study visits (every 1 to 2 weeks for nasal swab collection and approximately every 4 weeks for nasal swab and venous blood collection) and visits when COVID-like symptoms are present are still ongoing.

From 22 June 2021, HCW from CHBAH, not enrolled into the longitudinal cohort, who presented with COVID-like symptoms could enroll into a test negative case-control (TNC) study and be tested for SARS-CoV-2 infection by Nucleic Acid Amplification Test (NAAT) using a nasal swab. On 14 December 2021, this was expanded to two other Johannesburg hospitals: Charlotte Maxeke Johannesburg Academic Hospital (CMJAH) and Helen Joseph Hospital (HJH). HCW enrolled into the TNC could be present for multiple study visits. Enrolments are still ongoing at the three hospitals. HCW in the longitudinal cohort who were investigated for symptomatic illness were also eligible for inclusion in the TNC study.

Symptoms considered to be consistent with COVID-19 included any of the following: fever/feeling feverish, cough, sore throat, rhinitis, myalgia, shortness of breath, acute gastroenteritis/vomiting/nausea, impaired sense of smell or taste, fatigue, or headache. If an HCW had multiple symptomatic study visits between 24 November and 31 December 2021, only the visit with a positive NAAT SARS-CoV-2 result was included, or the first symptomatic visit if there was no positive NAAT result.

Demographic, health, and behavioral questionnaires collected personal information including COVID-19 vaccination history; previous SARS-CoV-2 infection was determined by documented NAAT positivity in the cohort participants or self-reporting.

### 2.2. Laboratory Methods

Total nucleic acids were extracted from nasal swabs using an automated NucliSENS-easyMAG nucleic acid extraction platform. NAAT was performed using the TaqPath COVID-19 diagnostic test from ThermoFisher that uses a triple-target (orf1ab, N gene, spike gene) design. Results were classified as positive for SARS-CoV-2 when the three targets or when both orf1ab and N gene had cycle threshold (Ct) values < 37 and inconclusive if only one target was detected with Ct values < 37. Results were classified as Omicron variant when orf1ab and N gene were detected but not the spike gene (SGTF).

Serum or plasma samples were collected at approximately monthly intervals from the longitudinal cohort participants. Participants enrolled into the TNC study had blood samples collected at enrolment. SARS-CoV-2 full-length spike protein immunoglobulin G (IgG) was measured by a quantitative assay on the Luminex platform as described [7,8]. The assay was evaluated for detection of antibodies against SARS-CoV-2 using COVID-19 convalescent plasma panel NIBSC 20/118. Based on differences in IgG titers from pre-COVID-19 and baseline samples when compared to post-infection samples of participants who were SARS-CoV-2 NAAT positive, 32 binding antibody units (BAU)/mL was selected as the threshold value indicative of seropositivity for full-length spike.

### 2.3. Statistical Analysis

Participants’ categorical characteristics were described as percentages and compared between NAAT-confirmed Omicron infected and uninfected HCW by Chi-square test. Continuous variables were represented as means with standard deviations (SD) or median with interquartile range (IQR) and compared by Student’s *t*-test or Mann–Whitney test, respectively. The association between Omicron infection and vaccination or previous SARS-CoV-2 infection was estimated by univariate and multivariate logistic regression. Participants were considered fully vaccinated if they received at least one dose of Ad26.COV.2 vaccine ≥14 days before the symptomatic visit or two doses of BNT162b2 vaccine, with the last dose ≥14 days before the symptomatic visit. Prior SARS-CoV-2 infections were categorized under the three previous pandemic waves: April to October 2020, November 2020 to April 2021, and May to September 2021. Differences in geometric mean units for spike IgG between NAAT-confirmed Omicron infected and uninfected HCW were analyzed on log_10_-transformed data.

A recursive partitioning approach was performed in the form of conditional inference tree. This method is particularly good at finding conditional thresholds in covariates by means of significance tests. Significance tests of the null hypothesis, that Omicron infection risk is identical on either side of the proposed threshold, are performed at each node of the tree in a recursive way. These tests are done by means of the conditional distribution of linear statistics in the permutation test framework, as outlined by Hothorn et al., which for two categorical variables corresponds to a Chi-square test [9]. Recursion is stopped when the obtained Bonferroni-adjusted *p*-values meets the user input significance level (in this case 90%). The final tree outlines all the splits for which the null hypothesis was rejected, i.e., for which a difference in outcome is statistically significant with 90% confidence across merging branches. With this type of conditional partitioning, a training set can be used to find the relevant splits, after which a validation portion of the data can be used to evaluate the method’s predictive power, in particularly type I errors (this method predicts the outcome to be a symptomatic infection, when in reality, none was recorded for that person) obtained with this method [9]. This analysis was performed using the rpart, party, and caret R packages.

For the purposes of the analyses presented throughout, serological measurements were restricted to blood samples collected within 3 months of symptomatic visits. If multiple samples were available, the sample collected closest to the symptomatic visit was used. Participants were excluded from the serology analysis if they received any vaccine between the last blood draw and the symptomatic visit or if the last blood draw was <14 days after the last vaccination. Since IgG antibodies after natural infection among vaccinated and previous infected individuals can rise quickly, we did a sub-analysis excluding the samples collected on the day of the symptomatic visit.

## 3. Results

The first case of SARS-CoV-2 with SGTF among HCW in our study was detected on 24 November 2021, prior to which the last confirmed SARS-CoV-2 infection was on 20 September 2021. From 24 November to 31 December 2021, 445 HCW had at least one symptomatic visit where nasal swabs were collected and tested by Taqpath NAAT for SARS-CoV-2 infection. Nine HCW had inconclusive results and were excluded from the analysis, and all but three (also excluded from the analysis) of the SARS-CoV-2 infections detected during this period had NAAT results with SGTF and putatively were Omicron variant.

Among the 433 symptomatic HCW included in the analysis, 190 (43.9%) had a positive NAAT. There were no reported Omicron infection-related hospital admissions among the study participants. Overall, 270 (62.4%) received a single dose of the Ad26.COV.2 vaccine (median 280 days; interquartile range (IQR): 257, 287), and 49 (11.8%) received a booster dose (median of 22 days; IQR: 18, 33) ≥14 days before the symptomatic visit. Only 26 (6.3%) HCW received two doses of BNT162b2 ≥ 14 days before the visit, and 69 (16.7%) were unvaccinated. Vaccination coverage was similar among HCW with symptomatic illness in whom Omicron was and was not identified (Table 1). Additionally, there was no difference in the odds of identifying Omicron between unvaccinated and vaccinated HCW, although the numbers for BNT162b recipients were low (Table 2).

Overall, 154 (35.6%) HCW had at least one SARS-CoV-2 NAAT-confirmed infection prior to November 2021, 53 (34.4%) of whom were reinfected with Omicron, compared with 137 Omicron infections among the 279 (49.1% *p* = 0.003) HCW without previous NAAT-confirmed infection. Participants with previous NAAT-confirmed infection had lower odds of Omicron infection compared with those without past infection (adjusted odds ratio (aOR) 0.55, 95% confidence interval (CI): 0.36, 0.84). Stratifying by timing of previous infection, infection during the preceding third wave was associated with lower odds of symptomatic Omicron illness relative to HCW without any previous NAAT-confirmed infection (aOR 0.40, 95%CI: 0.20, 0.80); likewise, individuals who were infected during the second wave had similar lower odds of being infected with Omicron during the study period (aOR 0.49, 95%CI: 0.20, 1.23), although not significant (Table 2).

Anti-spike IgG geometric mean units (measured in 267 participants) were lower in HCW who eventually had an Omicron infection compared with those who never tested positive (577 binding antibody unit (BAU)/mL, vs. 968 BAU/mL, *p* = 0.009) (Table 1). Excluding blood samples collected at the time of the current visit, a similar trend in IgG levels was observed (Table 1).

**Figure 1:**
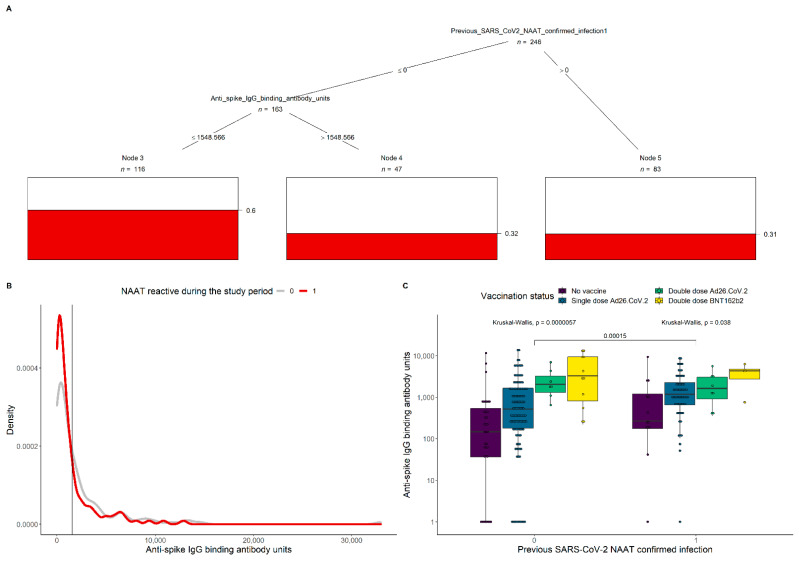
Conditional inference of Omicron infection probability and anti-spike IgG levels by prior SARS-CoV-2 NAAT-confirmed infection. (**A**) Inferred significant splits in previous SARS-CoV-2 NAAT-confirmed cases and spike IgG levels impact on the probability of having an Omicron infection during the study period (indicated by the red bars). The tree was generated from a training set composed of 90% of all visits with a known serological result. The algorithms infection predictive power was measured to be 72% in the remaining 10% of the data, with 23% type I error. (**B**) Antibody density distributions for participants with either NAAT-confirmed Omicron infection (red line) or no infection (grey line) during the study period. The vertical black line corresponds to the threshold 1549 anti-spike IgG binding units that emerged from the analysis on panel A. (**C**) Anti-spike IgG levels by prior SARS-CoV-2 NAAT-confirmed infection and vaccination status. Kruskal–Wallis tests indicate significant differences in IgG concentrations between participants with different levels of prior infection (*p* = 0.00015), and with more doses of vaccination in those who were not previously exposed (*p* = 0.000057). A lower significance in differences in IgG concentration for groups with different vaccination status for those who had a prior confirmed SARS-CoV-2 infection (*p* = 0.038).

To further investigate which combinations of covariates significantly modulate Omicron infection, a conditional inference tree was built (Figure 1A). Significance was detected in previous SARS-CoV-2 NAAT-confirmed cases and those with spike IgG levels > 1549 BAU/mL (Figure 1B), each with only 33% probability of infection. The boxplots in Figure 1C represent the anti-spike IgG levels by prior SARS-CoV-2 NAAT-confirmed infection and vaccination status. Overall, IgG concentrations were higher among HCW with prior infection (*p* = 0.00015), and in the group not previously infected in those with more vaccine doses (*p* = 0.000057). A lower significance was detected among the groups with different vaccination status for those who had a prior confirmed SARS-CoV-2 infection (*p* = 0.038).

## 4. Discussion

We show that prior SARS-CoV-2 infection prevented symptomatic reinfection with Omicron at 45–60%, which is consistent with results from a large population study in Qatar [10]. Although it has been suggested that Omicron is evasive to neutralizing antibodies induced by natural infection from previous variants or vaccine-elicited [11], we show that HCW who were not infected by Omicron during the five week period of this analysis had higher concentration of anti-spike IgG prior the symptomatic visit, with >1549 BAU/mL being the threshold suggestive of significant reduction in the risk of symptomatic Omicron infection. This might be due to some residual neutralization activity, or that anti-spike IgG recognizes the virus beyond neutralization via Fc-effector mechanisms, as recently suggested [12]. Protection can be achieved by prior infection (irrespective of the vaccination status) or by recent vaccination, with participants who were unvaccinated or those who received a single dose of Ad26.COV.2, most of whom more than eight months prior, showing significantly lower levels of anti-spike IgG. Although we did not evaluate cell-mediated immunity and immune memory, it has been reported that after natural infection, the T-cell-mediated responses are targeted across a larger variety of epitopes than the humoral response, and therefore, might be more durable to genetic changes in viral epitopes [13]. Moreover, studies showed that the majority of CD4+ and CD8+ T-cell response to the spike protein induced by vaccination or prior natural infection cross-recognized the Omicron variant, thereby likely contributing to protection against severe disease [14,15]. In addition, Omicron-infected patients had similar T-cell responses to ancestral spike, nucleocapsid, and membrane proteins to those found in patients hospitalized in previous waves [15].

A limitation of our study was that prior infection was assessed by NAAT only, with some unvaccinated participants with no previous NAAT-confirmed infection being seropositive for anti-spike antibody, demonstrating exposure to SARS-CoV-2 before blood collection. A high SARS-CoV-2 seropositivity in South Africa prior to the Omicron wave has actually been suggested as a plausible explanation for the disconnection between hospitalization/death rates and infection rates associated with Omicron in the country [7]. Other limitations include the relatively small sample sizes of our groups and the fact that we did not perform any functional antibody assays, and as such, we cannot identify the exact protection mechanism.

We found high reinfection and vaccine breakthrough infection rates with the Omicron variant among HCW at three hospitals in Johannesburg, South Africa. Inherently, either natural infection or vaccination elicits immune responses that decays over time, with the specificity (cross-immunity), quality (neutralization), and magnitude (absolute amount) of circulating antibodies determining the likelihood of future symptomatic infections. Although a study from the United Kingdom showed limited protection against symptomatic Omicron illness after BNT162b2 or ChAdOx1 vaccination [16], recent results on the vaccine effectiveness of Ad26.COV.2 booster dose in South Africa against Omicron hospitalization also demonstrates the value of booster vaccinations [4].

## Figures and Tables

**Table 1:** Healthcare workers with at least one symptomatic study visit between 24 November and 31 December 2021.

	Symptomatic Study Visits 433		
	Omicron Infection *n* = 190 (43.9%)	NAAT Negative, No Omicron Infection *n* = 243 238 (56.1%)	*p*-Value
Race			0.68
Black-African	172 (90.5)	217 (89.3)	
Other	18 (9.5)	26 (10.7)	
Female	154 (81.5)	203 (83.5)	0.58
Mean age in years (SD)	37.4 (9.2)	38.0 (10.0)	0.50
Study site			0.005
CHBAH	156 (82.)	185 (76.1)	
HJH	15 (7.9)	9 (3.7)	
CMJAH	19 (10.0)	49 (20.2)	
No vaccine	32 (16.8)	37 (15.2)	0.72
Ad26.COV.2 single dose ^a^	121 (63.9)	149 (61.3)	
Ad26.COV.2 booster dose ^b^	21 (11.1)	28 (11.5)	
BNT162b2 ^c^	9 (4.7)	17 (7.0)	
Median time in days from 1st Ad26.COV.2 dose to visit (IQR)	280 (260, 287)	279 (257, 287)	0.70
Median time in days from Ad26.COV.2 booster dose to visit (IQR)	23 (18, 32)	22 (19, 35)	0.74
Previously SARS-CoV-2 NAAT-confirmed infection	53 (27.9)	101 (41.6)	0.003
Never infected	137 (72)	142 (58.4)	
1st wave ^d^	32 (16.8)	48 (19.8)	0.018
2nd wave ^d^	8 (4.2)	15 (6.2)	
3rd wave ^d^	13 (6.8)	36 (14.8)	
Previously infected > 1	0	2 (0.8)	
	*n* = 174 ^e^	*n* = 215 ^e^	
No Ad26.COV.2, no previous SARS-CoV-2 NAAT-confirmed infection	23 (13.2)	23 (10.8)	0.18
No Ad26.COV.2, previous SARS-CoV-2 NAAT-confirmed infection	9 (5.2)	14 (6.5)	
Ad26.COV.2, no previous SARS-CoV-2 NAAT-confirmed infection	99 (56.9)	104 (48.6)	
Ad26.COV.2, previous SARS-CoV-2 NAAT-confirmed infection	43 (24.7)	73 (34.1)	
	*n* = 123	*n* = 144	
Anti-spike IgG binding antibody units > 32/mL	113 (91.9)	134 (93.1)	0.71
Anti-spike IgG geometric mean units (95% CI)	577 (428, 780)	968 (755, 1242)	0.009
Mean time in days from blood collection to visit (SD)	6.6 (17.8)	8.2 (19.3)	0.47
Serology results excluding bloods collected at the time of the current visit	*n* = 28	*n* = 37	
Anti-spike IgG binding antibody units > 32/mL	25 (89.3)	35 (94.6)	0.64
Anti-spike IgG geometric mean units (95% CI)	511 (312, 836)	919 (575, 1468)	0.09
Mean time in days from blood collection to visit (SD)	29.1 (27.3)	32.3 (26.1)	0.64

Results are *n* (%) unless stated otherwise. CHBAH: Chris Hani Baragwanath Academic Hospital; HJH: Helen Joseph Hospital; CMJAH: Charlotte Maxeke Johannesburg Academic Hospital; SD: standard deviation; IQR: interquartile range; CI: confidence interval; NAAT: Nucleic Acid Amplification Test. ^a^ Received a single Ad26.COV.2 vaccine dose ≥ 14 days before visit. ^b^ Received a booster Ad26.COV.2 vaccine dose ≥ 14 days before visit. ^c^ Received two BNT162b2 vaccine doses, with second dose ≥ 14 days before visit. ^d^ 1st wave: April to October 2020, 2nd wave: November 2020 to April 2021, 3rd wave: May to September 2021. ^e^ Excluding participants who received any BNT162b2 vaccine or those receiving the Ad26.COV.2 vaccine < 14 days before visit.

**Table 2:** Protection against Omicron infection by vaccination or previous SARS-CoV-2 NAAT-confirmed infection.

	Omicron Infection	NAAT Negative, No Omicron Infection	Unadjusted OR (95%CI)	Adjusted OR (95%CI)
Never vaccinated	32	37	Reference	Reference
Ad26.COV.2 single dose ^a^	121	149	0.94 (0.55, 1.60)	0.81 (0.46, 1.43) ^e^
Ad26.COV.2 booster dose ^b^	21	28	0.87 (0.41, 1.81)	0.94 (0.44, 2.03) ^e^
BNT162b2 two doses ^c^	9	17	0.61 (0.24, 1.56)	0.59 (0.23, 1.57) ^e^
No previous SARS-CoV-2 NAAT-confirmed infection	137	142	Reference	Reference
Previous SARS-CoV-2 NAAT-confirmed infection	53	101	**0.54 (0.36, 0.82)**	**0.55 (0.36, 0.84) ^f^**
1st wave ^d^	32	48	0.69 (0.42, 1.15)	0.71 (0.42, 1.19) ^f^
2nd wave ^d^	8	15	0.55 (0.23, 1.35)	0.49 (0.20, 1.23) ^f^
3rd wave ^d^	13	36	**0.37 (0.19, 0.74)**	**0.40 (0.20, 0.80) ^f^**
No previous SARS-CoV-2 NAAT-confirmed infection, no vaccine	23	23	Reference	Reference
No previous SARS-CoV-2 NAAT-confirmed infection, Ad26.COV.2 single dose	85	91	0.93 (0.49, 1.79)	0.89 (0.46, 1.72) ^g^
No previous SARS-CoV-2 NAAT-confirmed infection, Ad26.COV.2 booster dose	14	13	1,06 (0.42, 2.79)	n.a
Previous SARS-CoV-2 NAAT-confirmed infection, no vaccine	9	14	Reference	Reference
Previous SARS-CoV-2 NAAT-confirmed infection, Ad26.COV.2 single dose	36	58	0.97 (0.38, 2.46)	0.82 (0.28, 2.44) ^g^
Previous SARS-CoV-2 NAAT-confirmed infection, Ad26.COV.2 booster dose	7	15	0.73 (0.21, 2.48)	0.55 (0.14, 2.09) ^g^
**Using antibody information and threshold from the conditional inference tree**				
No previous SARS-CoV-2 NAAT-confirmed infection, no vaccine, IgG < 300 ^h^	18	15	Reference	Reference
No previous SARS-CoV-2 NAAT-confirmed infection, Ad26.COV.2 single dose, IgG > 1549 ^i^	34	63	**0.45 (0.20, 1.00)**	**0.42 (0.18, 0.85) ^g^**
No previous SARS-CoV-2 NAAT-confirmed infection, Ad26.COV.2 booster dose, IgG > 1549 ^i^	10	11	0.76 (0.25, 2.27)	n.a

OR: Odds Ratio; CI: Confidence Interval; NAAT: Nucleic Acid Amplification Test. Numbers in bold mean significant OR. ^a^ Received a single Ad26.COV.2 vaccine dose ≥ 14 days before visit. ^b^ Received a booster Ad26.COV.2 vaccine dose ≥ 14 days before visit. ^c^ Received two BNT162b2 vaccine doses, with second dose ≥ 14 days before visit. ^d^ 1st wave: April to October 2020, 2nd wave: November 2020 to April 2021, 3rd wave: May to September 2021. ^e^ Adjusted for study site and having a previous SARS-CoV-2 NAAT-confirmed infection. ^f^ Adjusted for study site and vaccination. ^g^ Adjusted for study site. ^h^ Excluding participants with anti-spike IgG binding antibody units > 300/mL, as this antibody level was the lowest quartile among unvaccinated participants with prior SARS-CoV-2 NAAT-confirmed infection, and as such, a putative level for previous asymptomatic infections. ^i^ Excluding participants with anti-spike IgG binding antibody units < 1549/mL, as this was the threshold suggestive of a significant reduction in the risk of symptomatic Omicron infection among vaccinated participants without prior SARS-CoV-2 NAAT-confirmed infection.

## Data Availability

Individual participant data that underlie the results reported in this article, after deidentification will be shared upon request. Researchers who wish to use the data to address any specific questions not directly addressed under the study objectives and which the data would lend itself to, who provide a methodologically sound proposals that has been approved by an independent review committee, may request the data. Requests should be directed to marta.nunes@wits-vida.org.
